# Voluntary Medical Male Circumcision to Prevent HIV: Modelling Age Prioritization in Namibia

**DOI:** 10.1007/s10461-019-02556-y

**Published:** 2019-06-18

**Authors:** Peter Stegman, Bridget Stirling, Brad Corner, Melissa Schnure, Denis Mali, Ella Shihepo, Katharine Kripke, Emmanuel Njeuhmeli

**Affiliations:** 1 Project SOAR, Avenir Health, 7064 Eastern Avenue, NW, Washington, DC 20012 USA; 2 University of Calgary in Qatar, Doha, Qatar; 3 United States Agency for International Development, Windhoek, Namibia; 4 Project SOAR, Palladium, Washington, DC USA; 5 National AIDS Control Program, Ministry of Health and Social Services, Windhoek, Namibia; 6 United States Agency for International Development, Washington, DC USA

**Keywords:** Namibia, Sub-Saharan Africa, HIV/AIDS, HIV prevention, VMMC

## Abstract

Voluntary Medical Male circumcision (VMMC) has been part of prevention in Namibia since 2009. Yet, as of 2013, VMMC coverage among 15- to 24-year-olds was estimated at less than 22%. Program data suggests uptake of VMMC below age 15 is lower than expected, given the age distribution of the eligible population. Nearly 85% of VMMCs were for males between ages 15 and 29, while boys 10–14 years were referred outside the program. This analysis uses the Decision Makers Program Planning Tool to understand the impact of age prioritization on circumcision in Namibia. Results indicate that circumcising males aged 20–29 reduced HIV incidence most rapidly, while focusing on ages 15–24 was more cost effective and produced greater magnitude of impact. Providing services to those under 15 could increase VMMC volume 67% while introducing Early Infant Medical Circumcision could expand coverage. This exercise supported a review of VMMC strategies and implementation, with Namibia increasing coverage among 10- to 14-year-olds nearly 20 times from 2016 to 2017.

**Electronic supplementary material:**

The online version of this article (10.1007/s10461-019-02556-y) contains supplementary material, which is available to authorized users.

## Introduction

A country of just 2.5 million people, Namibia has made progress over the past decade in reducing the spread of the human immunodeficiency virus (HIV) and death from acquired immune deficiency syndrome (AIDS). However, despite these efforts, HIV prevalence in Namibia is still among the highest in the world [1]. Several factors have been suggested as possible drivers of the high HIV prevalence including: multiple and concurrent partnerships; intergenerational sex; alcohol use; low condom use; transactional sex, population mobility; early age at sexual initiation and, central to this analysis, low coverage of male circumcision [2–5]. In addition, polygamy and multiple partnerships contribute as well [3]. There is a rapid rate of partner turnover among sexually active Namibians, especially male youths [6, 7].

The absence of circumcision in a population has been recognized as a risk factor for sexually transmitted infections (STIs) [8] including HIV [9, 10]. Circumcision, a one-time, preventative measure, partially protects HIV negative men from acquiring the virus [11–13]. In Namibia, the Voluntary medical Male Circumcision (VMMC) program was initiated in 2009. However, with little initial scale up, circumcision prevalence among males ages 15 to 49 only increased from 21% in 2007 [14] to 25.5% in 2013 [5] according to Demographic and Health Surveys (DHS). According to the population-based HIV impact assessment (PHIA) conducted in Namibia in 2017, VMMC coverage among 15 to 64-year-olds was 36.4% nationally for that year [15]. Modelled coverage for the same year using the DMPPT came out to be 31.9%. Although the data available from the PHIA did not report a confidence interval for its coverage number, it would be reasonable to assume that the model coverage is within the confidence interval for the survey coverage.

The President’s Emergency Plan for AIDS Relief (PEPFAR), the U.S. Government’s response to the global HIV/AIDS epidemic, has been in partnership with the Namibian government to address common priorities, including expanding coverage of the VMMC program. Between 2010 and 2014, PEPFAR supported 13,531 VMMCs in Namibia [16]. In 2015, there were 16,507 circumcisions conducted. There are significant barriers to VMMC provision in Nambia. Importantly, some areas of the country, particularly the northern regions (Omusati, Ohangwena, Caprivi, Oshana, and Zambezi) are not traditionally circumcising areas [2, 5]. As circumcision, even as a rite of passage, is not within their social context, promotion of VMMC is a constant challenge. Additionally, lower rates of circumcision correspond to certain demographic characteristics in the country, such as lower educational attainment, and lower wealth and material affluence [5]. Thus, socio-cultural, and socio-economic barriers to VMMC service delivery may also exist. Limited access to services may also contribute given that a large number of men work in mines, on large agricultural estates or ranches, and fishing boats, where they cannot leave to seek services and service provision may not be able to reach out to them.

There are even barriers to service provision that are age specific. Policy barriers, for instance, limit access to services for younger age groups (i.e. 10- to 14-years) who need permission from parents or guardians to be circumcised. Similarly, it was commonly understood that nurses were not allowed to perform a Dorsal Slit circumcision procedure, the current WHO policy for circumcising younger males, effectively cutting younger clients off from services provision, unless they could access private sector providers [2, 17]. In 2016, during the period of this modelling exercise, PEPFAR issued its Technical Considerations for VMMC that provided policy guidance that programs should initially prioritize service provision to males ages 15 to 29 [16]. The aim of this policy guidance was to enhance coverage of VMMC to at least 60% of eligible males ages 15 to 29 years to achieve the most immediate epidemiological impact. These Technical Considerations coincided with the release of the WHO/UNAIDS Policy Brief: A Framework for VMMC, which set a target for 90% coverage of VMMC for 10- to 29-year-olds [18]. In Namibia, according to the 2013 DHS, about 45% of people ages 15 to 19 years reported ever having sexual intercourse [5], while 5% of women and 10% of men who were 25 to 49 at the time of the 2013 DHS [5] reported having sex by age 15 suggesting that an effective VMMC strategy would try to target males before becoming sexually active.

In 2015, the Ministry of Health and Social Services (MOHSS) was interested in better understanding the impact of targeting different age groups for VMMC program service delivery in the country. In 2015/2016, the Decision Makers Program Planning Tool (DMPPT) Version 2.1 was applied in Namibia to explore the epidemiological impact and cost effectiveness of different age-prioritization strategies for VMMC service delivery. The results of the analysis supported program planning and enabled program managers and implementing partners to better assess progress towards their targets. This paper describes the process and results of the DMPPT modeling exercise in Namibia. It also lends some evidence to support future program decisions related to prioritization of age groups in the Namibian population.

## Methods

### Overview of DMPPT

This analysis used the DMPPT 2.1 model, which is described in more detail elsewhere [19]. It is a simple, deterministic, compartmental model based in Microsoft Excel designed to analyze the effects of age at circumcision on program impact and cost-effectiveness. The model breaks down the local male population into 5-year age bands and tracks the number of circumcised males in these groups over time, taking into account age progression and mortality. It then calculates discounted VMMC program costs and HIV infections averted in the population in each year of a user-specified VMMC scale-up strategy. These results are then compared with a baseline scenario in which the male circumcision (MC) prevalence remains the same. The baseline scenario assumes that traditional or other circumcisions that produced the baseline MC prevalence continue at the same rate as before the VMMC program was initiated.

### Data Used

The research team populated the DMPPT model with exogenous estimates of population size, mortality, and HIV prevalence and incidence projections from the spectrum/goals files for the country [20] that had been produced by program managers and experts in consultation with VMMC stakeholders. All DMPPT model inputs are available in Supplemental Table S1. Goals is a compartmental deterministic model within the Spectrum suite of models and incorporates demography, HIV epidemiology, sexual behavior, HIV disease progression, and the impact of HIV treatment and prevention interventions on mortality and new HIV infections. The Goals model had been calibrated to the national Spectrum AIDS Impact Model [20] HIV incidence estimates, which are based on national survey and sentinel surveillance data. Future HIV incidence projections were made using the Goals model and assumed full implementation of 90–90–90 treatment targets. Baseline circumcision prevalence by age group (i.e. circumcision prevalence prior to the establishment of the national VMMC program and scale-up of services) was derived from the 2006/2007 Demographic and Health Survey [14]. The total number of VMMCs performed in Namibia, disaggregated by age group, was based on PEPFAR program data for FY 2015. In addition, there were a limited number of VMMCs undertaken by the private sector, which were assumed to be included in the baseline circumcision rates and were not included in the analysis. This assumption is supported by data from the Population-based HIV Impact Assessment (PHIA) study, which show that a substantial proportion of circumcisions in Namibia are medical, even in provinces where there is no PEPFAR or Global Fund program.

The analysis used a unit cost of US$132 for VMMC. This cost was based on a recent facility-based costing study conducted in South Africa [20]. According to this study, labor costs accounted for $70.64. The cost for labor for other countries were multiplied by the ratio of each country’s purchasing power parity-adjusted per capita gross national income in 2014 [21] to that of South Africa. The other cost categories (consumables, continuous quality improvement, overhead, training, equipment, and vehicles) were assumed not to vary significantly across countries. Demand creation costs were not included in the unit cost. The resulting total country VMMC unit cost was increased by a nominal 15% to account for above-facility level costs, such as management and other programming [22]. Costs for antiretroviral therapy (ART) used a default of US$515 fully loaded per person, per year. This is based on an international weighted average median price in 2011 of $145 for first- and second-line ART drugs [23]; $222 for average service delivery and monitoring costs [23]; plus an additional 40% for costs above the facility level for administration, logistics, training, planning, and so forth [24]. This cost will need to be updated in the future.

### Analytical Framework

The results of the DMPPT modelling were assessed within an analytical framework to determine the potential impact and cost effectiveness of circumcising different client age groups. This framework examines four outcomes: (1) *immediacy* of impact, measured by how rapidly a reduction in societal level HIV incidence can be observed within a short-term, five-year implementation period; (2) *magnitude* of impact, which is measured by the extent of the overall reduction in societal level HIV incidence that can be observed after a longer 15 years of program implementation; (3) *efficiency*, as measured by the number of circumcisions required to avert a single HIV infection over 15 years; and (4) *cost effectiveness*, measured by the cost per each HIV infection averted after 15 years.

### Assessment of Impact

To isolate the impact of the provision of VMMC services to specific age groups, the research team created a series of artificial scenarios where coverage was scaled up for each individual five-year age group between 2015 and 80% target coverage in 2021. After 2021, target coverage was maintained and projected out to 2050 while keeping all other age groups at constant 2015 coverage. The authors then compared the decrease in HIV incidence for each age-specific scale-up scenario with the other age groups held at constant coverage to assess which age group achieved the greatest impact on societal level HIV incidence over a short-term of 5 years (immediacy of impact) and a long-term of 15 years (magnitude of impact).

The authors then modeled various VMMC scale-up strategies targeting specific combinations of age groups that are generally more relevant for program planning (e.g., 10 to 34, 10 to 29, 10 to 24, 15 to 34, 15 to 29, 15 to 24, and 10 to 49). For each of these scale-up strategies, a target coverage of 80% was set to be achieved by 2021. Increases in circumcision coverage to the target level were projected by applying a linear interpolation to the baseline circumcision prevalence for each age group combination in 2015 and the target coverage in 2021. From 2021 onward, the coverage was maintained at the target level. Modeling each scale-up strategy over a 15-year period between 2016 through 2030 allowed the research team to look at the total number of HIV infections averted; the number of circumcisions needed to avert one HIV infection; and the total cost of the VMMC program. Costs, numbers of circumcisions, and infections averted were all discounted at a rate of 3% per year.

To examine the effects of circumcising younger age groups (i.e. infants and 10- to 14-year-olds) the authors conducted a series of analyses. In the first analysis, the authors explored the potential increases to the overall number of circumcisions performed across age groups over time that could be expected by providing circumcision services to younger age groups. The potential increases were modeled and compared using different scale up strategies as in Table 1: (1) a base strategy where coverage among 10- to 34-year-olds is scaled up to 80% within five years and then maintained at that coverage into the future; (2) an intensive EIMC strategy where coverage among infants is also scaled up to 80%, along with 10- to 34-year-olds in the base coverage strategy, over the same period and maintained into the future; and (3) a mixed strategy where, in addition to the base strategy, coverage of infant circumcision is scaled up to 40% over the same time period and this coverage is maintained into the future.

**Table 1:** Modelling scenarios exploring potential increases in circumcision coverage by introducing EIMC into the national VMMC program

National VMMC program scale up strategies		
Scenario 1	Scenario 2	Scenario 3
Base strategy	Intensive EMIC introduction	Mixed scale up strategy
Targeting 80% VMMC coverage for 10- to 34-year-olds only for national program scale up over 5 years and maintained at 80% thereafter	Targeting 80% coverage of 10- to 34-year-olds as in the base strategy, and including a target of 80% EIMC coverage over the same time period	Targeting 80% coverage of 10- to 34-year-olds as in the base strategy, and including a target of only 40% EIMC coverage over the same time period

In a second analysis, the authors examined the potential impact of the PEPFAR age prioritization policy of focusing on circumcising 15- to 29-year-olds over the short-term (2015–2022) using the following three VMMC scale-up scenarios found in Table 2: (1) aggressive scale-up of circumcision for men ages 15 to 29 years and service delivery among boys ages 10 to 14 years sustained at 2016 levels; (2) service delivery sustained at 2016 levels among men ages 15 to 29 years and no service delivery among boys ages 10 to 14 years; and (3) very aggressive targeting of men ages 15 to 29 years, and aggressive targeting of boys ages 10 to 14 years. This analysis is treated in more detail elsewhere [25], and drawing from that paper, the team defined ‘aggressive’ scale-up as achieving numbers of VMMCs that were 50% above historical accomplishments, and ‘very aggressive’ as 100% above.

**Table 2:** Scale up scenarios examining the potential impact of the PEPFAR age prioritization policy

PEPFAR age prioritization policy scale up strategies		
Scenario 1	Scenario 2	Scenario 3
Aggressive scale up 15–29 years Sustained coverage 10–14 years	Sustained coverage 15–29 years No service delivery for 10–14 years	Very aggressive scale up 15–29 years Aggressive scale up to 10–14 years
VMMC coverage of 15- to 29-year-olds increased by 50% above 2016 levels; VMMC coverage to 10- to 14-year-olds unchanged from 2016 levels	VMMC coverage of 15- to 29-year-olds unchanged from 2016 levels; no VMMC services provided to 10- to 14-year-olds	Very coverage 15- to 29-year-olds increased by 100% above 2016 levels; VMMC coverage of 10- to 14-year-olds increased by 100% above 2016 levels

To assess the potential impact, cost, and cost-effectiveness of introducing EIMC, the authors conducted two further analyses, described in a previous article [26]. One analysis compared two scale up scenarios. One, a base scenario where coverage is scaled up to 80% among 10- to 34-year-olds between 2016 and 2020 and is maintained at 80% to 2050, and another maintaining the same scale up strategy of the first scenario but adding attainment of 80% coverage of EIMC within the same timeframe. Comparison of these two scenarios allowed the authors to analyze the differences between the total number of circumcisions required by the program to meet coverage targets, the number and percentage of infections averted, and the number of circumcisions needing to be performed to avert a single HIV infection. Finally, to determine the cost and cost effectiveness of introducing EIMC into the national VMMC program, the authors conducted a sensitivity analysis in which the costs for EIMC were set at 100, 50, and 25% relative to the costs of provision of adolescent and adult VMMC services for a scale up strategy where coverage among 10- to 34-year-olds is scaled up to 80% between 2016 and 2020 and is maintained at 80% to 2050, to which attainment of 80% coverage of EIMC within the same timeframe is added.

Finally, the authors undertook a post hoc analysis comparing the annual, age-disaggregated program data from 2015, when the initial DMPPT country application was carried out, to the end of 2017, the latest period for which there are program data, to explore the possible changes in policy and program planning for VMMC in Namibia, over time. The research team assessed VMMC coverage levels across all age groups to determine what changes age-specific coverage patterns may have occurred over the period, potentially as a result of the DMPPT modelling exercise.

## Results

### Immediacy and Magnitude of Impact

**Fig. 1:**
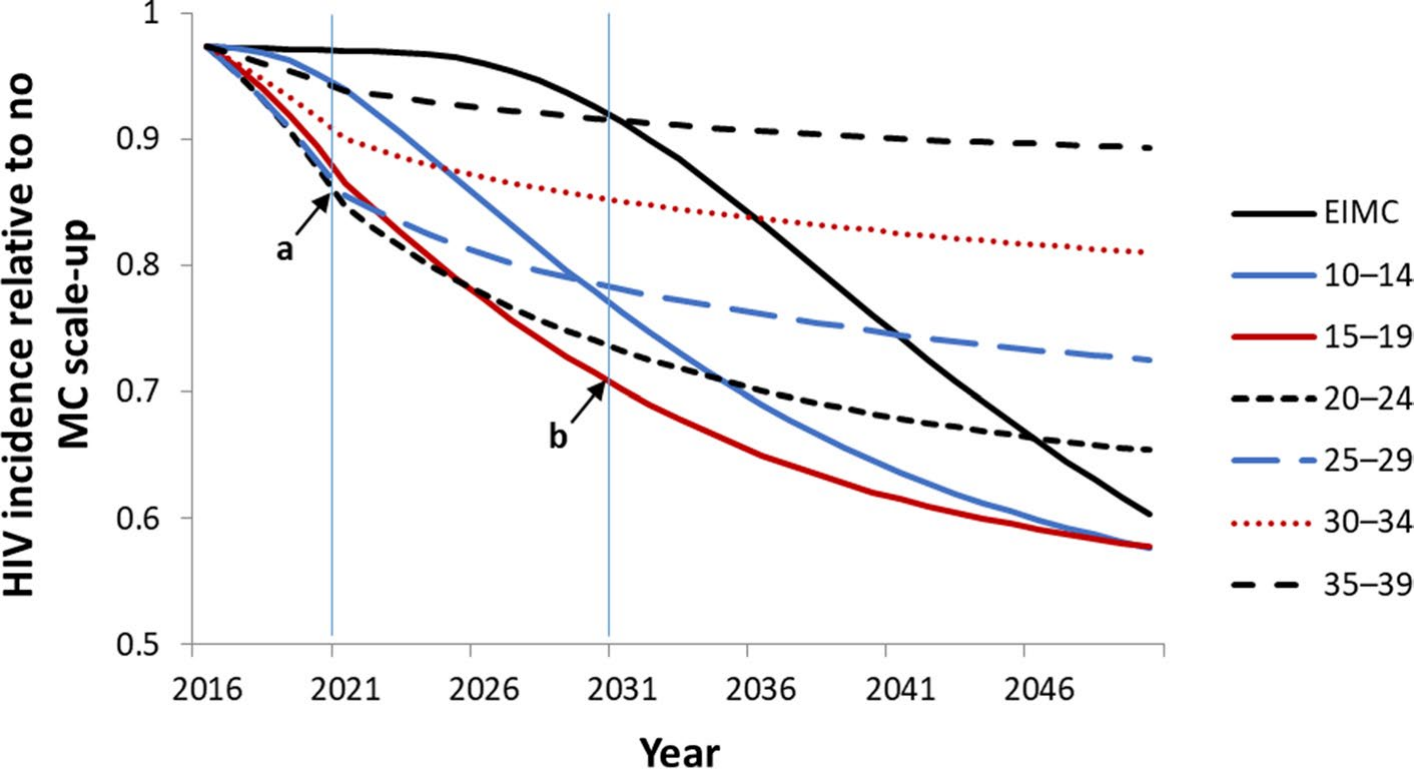
Relative reduction in HIV incidence for specific age groups compared with incidence in a population with no VMMC, 2015–2051

Adopting different age specific VMMC scale up strategies result in varied impacts on population levels of HIV incidence over the short and long terms. Figure 1 depicts the relative reduction in HIV incidence achieved by circumcising specific age groups compared with incidence in a population with no VMMC from 2015 to 2051. In these hypothetical scenarios, only males of the indicated age group are circumcised. The model shows that, over five years (point “a,” “immediacy of impact”), circumcising males ages 20 to 24, and 25 to 29 have the greatest impact on HIV incidence levels, and over 15 years (point “b,” “magnitude of impact”), circumcising males in the 15 to 19 and 20 to 24-year age bands will have the greatest impact. The period of 15 years (2016–2031, inclusive) was selected by a range of experts as it was considered long enough to discern appreciable impact in HIV incidence and short enough to be relevant to policymakers [19].

### Efficiency and Cost Effectiveness

**Fig. 2:**
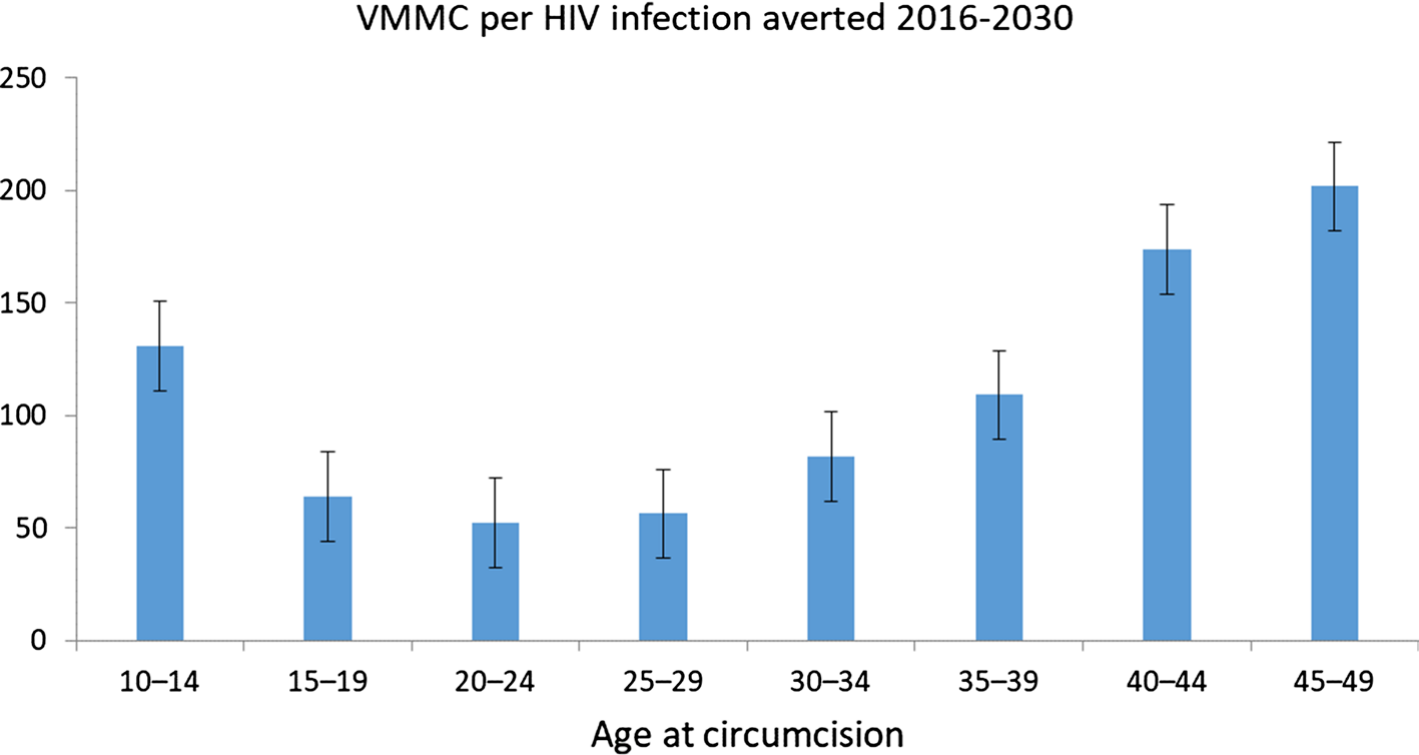
Number of circumcisions required to avert one HIV infection by age group in Namibia, over a 15-years period from 2016 to 2030

Efficiency in this analysis is defined as the number of circumcisions that would need to be performed in order to avert one HIV infection. Figure 2 presents the efficiency measures for each 5-year age group over a 15-year period in Namibia. Hypothetical scenarios were generated in which circumcisions are only performed on males of each specific five-year age group, and not broader groups inclusive of more than one five-year age band, with the infections averted then projected across the entire population. The figure indicates that the fewest circumcisions per HIV infection averted (IA) for the period (i.e. the most efficient), relative to the other strategies presented, would occur by circumcising 15- to 19-year-olds (63 VMMC/IA), 25- to 29-year-olds (56 VMMC/IA), and 20- to 24-year-olds (52 VMMC/IA).

**Fig. 3:**
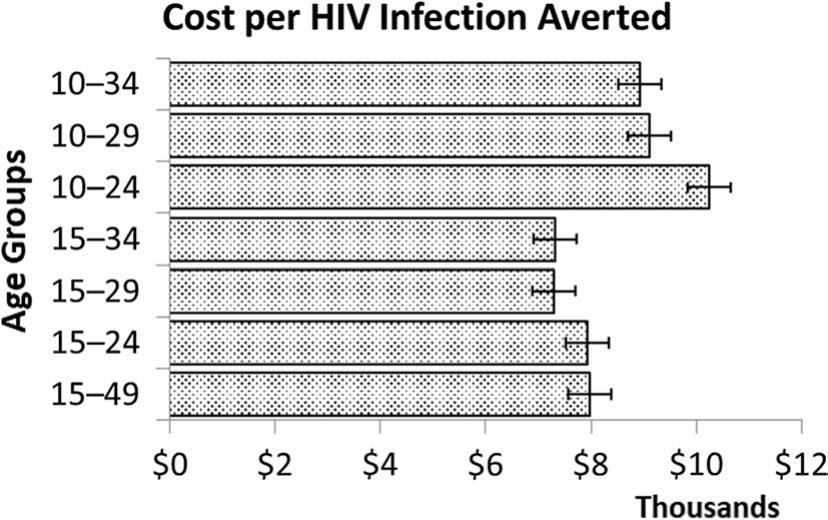
The cost per infection averted of different age grouping strategies in Namibia from 2016 to 2030

The research team next considered the cost effectiveness of the national VMMC program in Namibia by comparing several different age prioritization strategies that may be adopted over the period 2016–2031. In this analysis, cost effectiveness was defined as the relative discounted cost per HIV IA, measured over a period of fifteen years, and assessed the cost-effectiveness of circumcising age groups broader than five years. Figure 3 demonstrates that the most cost-effective strategy (i.e. the lowest cost per HIV infection averted), relative to the other strategies presented, would be one that circumcises the age group 15 to 29 years, which reflected Namibia’s VMMC strategy at the time, or 15 to 34 years, each cost $6200/HIA. The cost effectiveness of strategies aimed at circumcising age groupings that were slightly narrower (15- to 24-years-old) or broader (15- to 49-years-old) were only incrementally more expensive, at $6700 and $6800 per IA, respectively. According to the model, circumcising males before age 15 is less cost effective as the majority of this age group are not yet sexually active. Therefore, there is a delay for any benefits on expenditure for VMMC service provision for this age group.

### Circumcising Infants and Young Boys

**Fig. 4:**
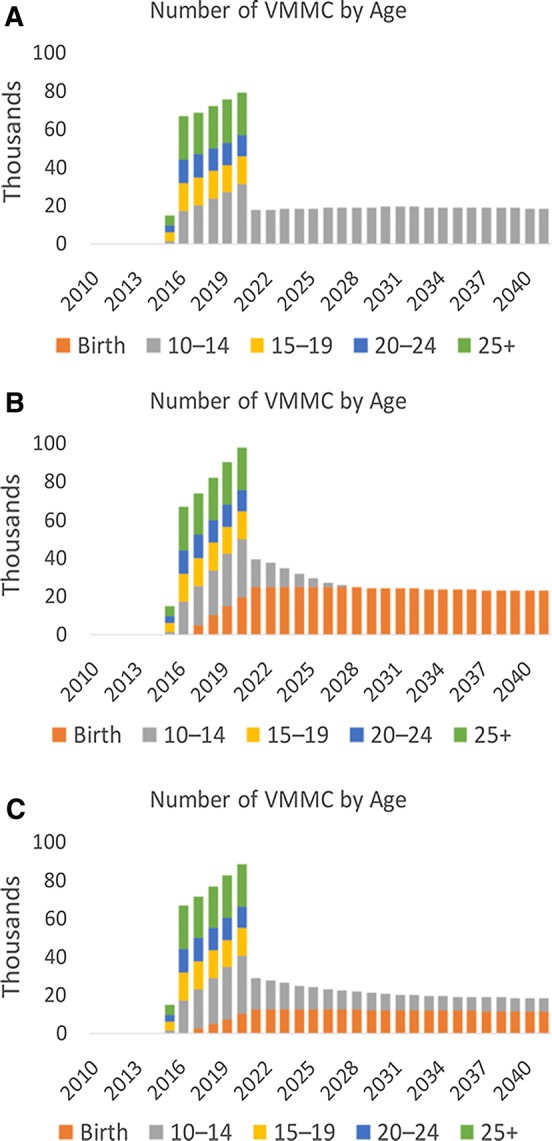
Three VMMC scale-up scenarios: **a** the number of circumcisions needing to be performed, by age group to achieve a scale up target of 80% coverage among 10- to 34-year-olds only; **b** the number of circumcisions needing to be performed, by age group to achieve a scale up target of 80% coverage among 10- to 34-year-olds + scale-up of EIMC to 80% coverage; **c** the number of circumcisions needing to be performed, by age group to achieve a scale up target of 80% coverage among 10- to 34-year-olds + scale-up of EIMC to 40% coverage

To further inform the Ministry of Health and Social Service’s VMMC programing, the authors analyzed the potential benefits of incorporating EIMC and circumcision of young boys ages 10 to 14 years into the existing VMMC strategy. With the limited performance of the VMMC program in the years leading up to this application of the DMPPT 2.1, the analysis focused primarily on what adopting a strategy that introduces EIMC might achieve in terms of expanded coverage across age groups. While this analysis is treated in more detail elsewhere [26], Fig. 4 presents the three scenarios modelled to depict the coverage of various strategies to introduce EIMC into Namibia’s national VMMC program. The graphs show the estimated number of circumcisions that would have to be performed in each age group for the different scale-up scenarios starting in 2016. As the research team was not provided any historical program data (prior to 2015) the graphs do not depict circumcisions performed during this period.

Graph A in the figure represents a national VMMC scale-up strategy increasing coverage to 80% for 10- to 34-year-olds over five years and then sustaining it into the future. In this scenario, to reach target coverage by 2021 would require significant increases in annual VMMC service delivery of around 4- to 5-fold compared with 2015 program accomplishments. After the target year is reached, VMMC coverage is maintained by continuing to circumcise approximately 18,000 10- to 14-year-olds every year. Graph B builds on the strategy presented in Graph A but introduces EIMC into the national VMMC program and scales-up coverage to 80%. This approach would require a four- to six-fold increase in the annual number of VMMCs performed, until 2021, to reach the coverage target, amounting to well over 300,000 circumcisions over the five-year period. Sustaining this strategy would necessitate the circumcision, on average, of 24,000 infants annually. Graph C in the figure presents a strategy where EIMC is scaled up to a coverage of 40%. As with the other strategies, the same initial push of increasing the annual number of VMMCs performed by 4- to 5-fold is required here as well, until the target date of 2021. The total number of circumcisions required to reach set coverage is just over 330,000. Into the future, these would be maintained by conducting around 20,000 circumcisions annually, with a mix of around 60% infants and 40% 10 to 14-year-olds.

### PEPFAR Age Prioritization Strategy

In 2016, PEPFAR issued new technical considerations for VMMC [27] that outlined a new strategic vision aimed at achieving the most immediate population-level impact from national VMMC programs. Such an impact would require a move from providing VMMC services to men ages 15 to 49 years to a narrower focus on circumcising males ages 15 to 29 years and scaling up to 60% coverage or more. Of course, promotion of such an implementation strategy is not meant to preclude the provision of circumcision services to males of other age groups, including boys 10- to 14-years old. This new policy direction from PEPFAR indicated that once the goal of reaching 60% coverage in the 15- to 29-year age groups had been reached, service provision to 10- to 14-year-olds could increase. Table 3 presents three VMMC coverage scenarios for Namibia that examines the effect of this policy guidance.

**Table 3:** Summary of three scenarios presenting VMMC coverage addressing PEPFAR age prioritization policy over a five-year period

PEPFAR policy standard	15–29 coverage start 2016 (%)	15–29 coverage start 2022 (%)	Increase in 15–29 coverage (%)
Scenario 1			
Aggressive VMMC provision for 15–29 and constant VMMC provision for 10–14	23	45	22
Scenario 2			
Constant VMMC provision for 15–29 and no VMMC provision for 10–14	23	39	16
Scenario 3			
Very aggressive VMMC provision for 15–29 and aggressive VMMC provision for 10–14	23	50	27

Scenario 1 represents the coverage that would be attained by strictly following the PEPFAR age prioritization. In this scenario, provision of VMMC to 15- to 29-year-olds is “aggressively” scaled up 50% above historical accomplishments, while circumcisions among 10- to 14-year-olds remain at 2015 levels. Net gains in coverage of 15- to 29-year-olds amounts to 22% point increase over 5 years. In comparison, Scenario 2, which keeps annual VMMC service provision to 15- to 29-year-olds constant and does not provide VMMC services to 10- to 14-year-olds, there is only an increase in coverage of 16% points among 15- to 29-year-olds over five years. Scenario 3 represents a more aggressive scenario and demonstrates what can be achieved if 15- to 29-year-olds are very aggressively circumcised (i.e., achieving numbers of VMMCs that were double the historical accomplishments), and if 10- to 14-year-olds are also targeted aggressively (achieving annual numbers of circumcisions that were 50% above historical accomplishments). In such a scenario, the National VMMC program in Namibia would be able to increase coverage among 15- to 29-year-olds by 27% points in 5 years and to a coverage of 50% at the end of 2021.

Table 4 presents a comparison of two VMMC scale up scenarios from 2016 to 2050 to assess the impact of adding EIMC to the national VMMC program in Namibia. In both scenarios, the VMMC scale up strategy is assumed to target expanding coverage among 10- to 34-year-olds only, while the other adds the introduction of EIMC. Scale up for each scenario is to reach 80% coverage within five years starting in 2016 and then maintaining that through to 2050, so as to ensure the timeframe of the model is sufficiently long to capture the impact of infant circumcision.

**Table 4:** The impact of adding EIMC to the national VMMC program from 2016 to 2050

Impact of adding EIMC			
	10–34 years	10–34 + EIMC	% Increase
Infection averted	26,000	28,000	7.6
Number of MCs	0.9 million	1.2 million	33.0
% Infection averted	34%	36%	2.0
VMMC per infection averted	39	49	25.6

According to the analysis, adding EIMC to the national program, and scaling up to 80% coverage, increases the overall number of circumcisions needing to be performed over the period by 300,000 or 33% from a scale up strategy targeting 10- to 34-year-olds only. The number of infections averted over the period increases by 2000 or 7.6%, translating to an increase of 2% in new HIV infections averted. The number of VMMCs needing to be performed to avert a single infection experiences a 25.6% increase from 39 to 49. This is largely accounted for by the increase in the overall number of circumcisions the program needs to perform in order to achieve the coverage target, and the delay in sexual debut among circumcised infants.

An analysis of the cost and cost-effectiveness of adding EIMC to the national VMMC program is presented in Table 5. The authors conducted a sensitivity analysis looking at the effect on overall cost and cost-effectiveness when the costs for EIMC were set at 100, 50, and 25% of the costs of provision of adolescent and adult VMMC services.

**Table 5:** The cost and cost-effectiveness of introducing EIMC to the scale up of the national VMMC program over the period 2016–2050

Cost and cost-effectiveness of adding EIMC			
	10–34 years	10–34 + EIMC	% Difference
EIMC cost at 100% of adult VMMC cost			
Total cost	$122 million	$156 million	25
Cost per infection averted	$5163.25	$6527.22	23
EIMC cost at 50% of adult VMMC cost			
Total cost	$122 million	$106 million	− 14
Cost per infection averted	$5163.25	$4606.79	− 11
EIMC cost at 25% of adult VMMC cost			
Total cost	$122 million	$81 million	− 40
Cost per infection averted	$5163.25	$3646.57	− 34

As the data suggest, when the cost of EIMC is 100% of the cost of adolescent and adult VMMC, there is an increase of 25% in the total cost and 23% in the cost per infection averted if a target of 80% EIMC coverage is added to the national program over the period 2016 to 2050. However, if the cost of EIMC compared to adolescent and adult VMMC begins to fall, the model predicts more beneficial outcomes. For example, when the cost of EIMC if estimated at 50% of VMMC services, the total cost of the program, despite the increase in numbers being circumcised, actually decreases by 14%. If the cost of EIMC is estimated even lower, at 25% the cost of VMMC services, the total cost of the national program decreases by 40% relative to the total number being circumcised. Cost of EIMC also has implications for the effectiveness of the national program. With the cost of EIMC at 50% of VMMC, there is a decrease in cost per infection averted from $5163 to $4606. A further reduction in the cost of EIMC makes the program more effective reducing the cost per infection averted to $3646.

### VMMC Program Gains

**Fig. 5:**
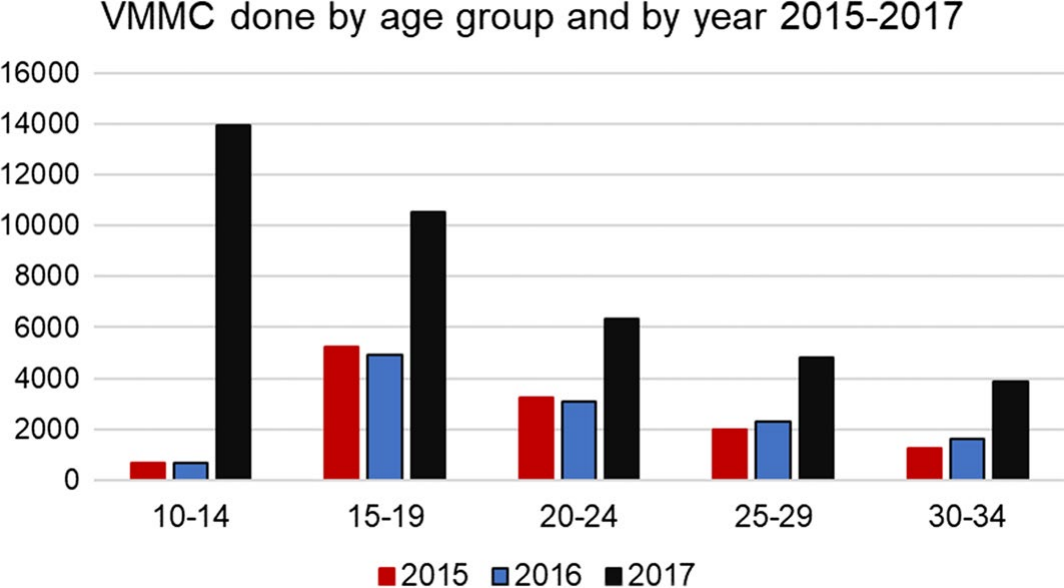
VMMCs performed by year in the age groups 10 to 34 years

After a year of VMMC program implementation in Namibia following our initial modelling analysis, the authors assessed any changes that occurred in the annual number of VMMC performed by age group. Figure 5 presents a comparison of VMMCs performed by age group and year for 2015–2017, with 2017 being the first year after the modeling exercise was conducted. The program data show a substantial increase in VMMCs performed across all age groups from 10 to 34 years. This increase is most dramatic in the age group 10 to 14, where there was more than a 20-fold increase in clients circumcised from 2016 to 2017. In the 15- to 19-year age group, productivity more than doubled over the same period. A doubling of circumcision volumes also occurred in the 20 to 24, 25 to 29, and 30 to 34-year age groups.

## Discussion

Programs for HIV prevention and treatment need to be as targeted and efficient as possible to maximize the benefits accrued from the resources allocated. This has placed increased pressure on countries and specific programs to use evidence for strategic planning. The modeling analyses in this paper demonstrate how adopting different age-targeted scale-up strategies can affect the overall impact, cost effectiveness, and efficiency of the national VMMC program in Namibia.

An important contribution of this analysis has been to highlight the programmatic benefits of circumcising younger age groups within VMMC scale up strategies in Namibia. The most immediate impact, in terms of HIV reduction, would be achieved by circumcising males ages 20 to 24 and 25 to 29 years, just prior to the ages of peak HIV incidence; the greatest overall impact over the next 15 years would occur as a result of circumcising 15 to 19 and 20 to 24-year-olds. However, it is worth noting that the benefits of circumcising younger males, especially infants, would be delayed by as much as 15 years. If scenarios were modeled beyond the 15-year time horizon, reductions in HIV incidence from circumcising younger males would potentially be much larger as these individuals would enter sexual maturity already being circumcised.

Circumcising males ages 15 to 29-years represents the most efficient and cost-effective scale-up strategy with a range of 52 to 63 circumcisions to avert one HIV infection, and a cost of $6200 per HIV infection averted. Cost per infection averted is substantially higher when including younger males in the age group 10 to 14-years because it takes longer for the benefits of circumcision to accrue due to this sub-population generally not having reached the age of sexual debut. Thus, while there is a delay in the societal level impact of nearly two decades, the impact is significant, and surpasses that achieved through any other age prioritization strategy. This is important when considering the sustainability of impact over the longer term. The model assumes achievement of the 90-90-90 goals, but VMMC impact and cost-effectiveness would be improved if Namibia does not reach the treatment targets by 2020 as outlined in the model.

According to these analyses, actual uptake of circumcision among males under age 15 was lower than would be expected prior to 2017, based on the age distribution of the eligible population and patterns observed in other countries. VMMC program data indicated lower than expected numbers of 10 to 14-year-olds undergoing VMMC in Namibia. According to MOHSS personnel and implementing partners (IPs), this was due, in large part, to two reinforcing factors: (1) a strict observation of PEPFAR guidance on age prioritization; and (2) a misinterpretation of MOHSS policy discouraging nurses from performing the dorsal slit circumcision method that is recommended by the World Health Organization for younger boys [17, 27]. As a result, no government nurses were offered training in this method and a practice was adopted to refer boys ages 10–14 years to alternative service providers when they came into government VMMC sites. Through this modelling exercise, however, dialogue was reopened around the policy, and with a revised interpretation, and training in dorsal slit now being offered to nurses, the numbers of 10- to 14-year-olds being circumcised dramatically increased [17].

Taking advantage of the relatively high levels of demand for services demonstrated by adolescents aged 10–14 would contribute to increased VMMC coverage among males aged 15 to 29. According to the 2013 Namibia DHS [5], more than 56% of males in the age group 15 to 19 have never had sexual intercourse, whereas that figure falls to only around 8% by the time they reach the 20 to 24-year age group. Epidemiologically, therefore, the most advantageous strategy would be to target males before they are sexually active. With HIV incidence highest among men aged 20 to 29, providing circumcision services to younger adolescents would potentially maximize infections prevented. Additionally, adolescents aged 10 to 14 represent a significant proportion, about 30%, of all men in the target ages of 15 to 29 years [5]. Targeting the 10- to 14-year-old age group is likely to be more cost effective as there is already demand for services, and younger adolescents do not require complex and extensive demand creation efforts. These seemingly easy efforts should help Namibia’s national VMMC program to address the WHO/UNAIDS strategy of increasing VMMC coverage among 10- to 29-year-olds to 90% by 2021 [18].

While focusing service provision on younger clients has the potential to increase overall coverage of circumcision, the inclusion of EIMC into the implementation of the national VMMC program offers a likely strategic pathway to sustain those high levels of programmatic coverage. The impact of EIMC, however, is only slightly greater in terms of infections averted than pursuing a strategy that targets 10- to 34-year-olds only, and such a benefit may not be worth the increased investment required to perform 33% more circumcisions. A greater impact from EIMC comes from its effect of decreasing overall program cost, relative to the numbers of circumcisions performed, and the cost per infection averted. The challenge, however, is the limited data available on the unit cost of EIMC compared to VMMC. For this analysis, it is assumed that EIMC costs less than adolescent and adult VMMC. This assumption is based on a number of considerations such as EIMC being a simpler procedure compared to VMMC, which could be easily implemented as an integrated program within existing antenatal clinics, rather than a standalone service as VMMC is provided. However, country programs will have to assess the available data in order to make the strategic decision that fits the prevailing context and environment.

## Study Limitations

While the results from the modelling exercises can provide estimates that assist with program decision-making, it is important to note that these estimates are not meant to be the only information on which strategic direction is ultimately determined. Numerous other considerations may have an impact, such as policy goals, funding constraints, human resources or infrastructural limitations, cultural or policy barriers, etc., and these will need to be weighed together with any modelling results.

HIV incidence estimates in the model are drawn from spectrum and are higher in the younger age group 15 to 24 years than was measured in the NAMPHIA study. This would tend to overemphasize the impact and cost-effectiveness of this age group compared with older age groups. In addition, while the spectrum incidence estimates for 15 to 49 years and the 15 + age grouping were within the confidence interval of the PHIA measurements, they were higher than the point estimates. Therefore, the overall impact estimates coming out of spectrum may be higher than the actual impact. These discrepancies will not have any effect on the coverage estimates. It should be noted that, when the analyses based on the DMPPT modelling were conducted, the PHIA data were not available for us to calibrate the Spectrum files to them.

The foregoing analysis was constrained by the lack of historical circumcision data prior to 2015, which limited the ability to project the impact of the VMMC program in Namibia. Application of the DMPPT in countries is closely guided by the country team’s assessment of the program and their analytical preferences. Stakeholders during the DMPPT country application in Namibia, voiced little confidence in the circumcision figures before 2015. At the time of this analysis, due to historically low program performance, it was thought that there was little value added in going through a process to validate the number of circumcisions done in previous years to add to the model. It was decided that the model should start at 2015, using circumcision numbers with which the country team was more comfortable. However, not having historical data was a limitation in that introduced a risk that projections of circumcisions required to reach coverage targets, impact, cost, and cost-effectiveness may be less accurate, depending on the scale of the missing numbers. Similarly, there was a gap in data on the numbers of 10- to 14-year-olds being circumcised. The research team was informed by program implementers that 10- to 14-year-olds that present for circumcision are referred for services provided in the private sector. However, these boys are not tracked once they have been referred, so there is little data on the number of circumcisions performed in this age group. Implementers in Namibia informed the research team that they did not believe these adolescents were actually accessing the private providers once referred.

As with any model, the DMPPT has some limitations, which are discussed in greater detail in another paper [19]. An additional limitation was that the model assumes the same unit cost per VMMC regardless of client age. Unit costs are likely to vary with the age of the client or the procedure used. In turn, the most cost-effective ages to prioritize, could likely be affected.

The analysis was further limited in not being able to determine VMMC coverage and set targets at the district levels as inadequate district-level data exists. Efforts are underway to generate more local level data that would facilitate more granular coverage estimates. Finally, there was little useable data addressing the issue of both internal and cross-border migration. What reports do exist, demonstrate a certain level of economic migration, but these data were not granular enough, nor were they mirrored by circumcision data coming from the MOHSS, to indicate migration’s effect on circumcision numbers region to region.

## Conclusions

While progress has been slow in attaining high VMMC coverage across all age groups in Namibia, this modelling analysis, using the DMPPT 2.1, was able to provide important data for programmatic decision making. The presentation of this analysis provided the opening for much-needed dialogue around how best to reorient and manage the program into the future. Key among the considerations made in this analysis was the need for rapidly scaling up the VMMC program, and the contribution of circumcising younger age groups, especially 10- to 14-year-olds. There continues to be discussion around the inclusion of EIMC into the existing strategy, but for the immediate term, by maximizing the existing service delivery capacity, and circumcising more 10- to 14-year-olds presenting for circumcision, Namibia has been able to make substantial progress.

## Electronic Supplementary Material

Below is the link to the electronic supplementary material.

Supplementary material 1 Inputs to the DMPPT. This table provides the inputs used in the DMPPT for the age prioritization analysis in Namibia. (XLSX 89 kb)
